# The effectiveness of adapted group mindfulness-based stress management program on perceived stress and emotion regulation in midwives: a randomized clinical trial

**DOI:** 10.1186/s40359-022-00823-7

**Published:** 2022-05-13

**Authors:** Fatemeh Aghamohammadi, Omid Saed, Reza Ahmadi, Roghieh Kharaghani

**Affiliations:** 1 School of Nursing and Midwifery, Zanjan University of Medical Sciences, Zanjan, Iran; 2 Department of Clinical Psychology, School of Medicine, Zanjan University of Medical Sciences, Zanjan, Iran; 3 Department of Midwifery, School of Nursing and Midwifery, Zanjan University of Medical Sciences, Zanjan, Iran

**Keywords:** Midwives, Stress, Mindfulness, Emotion regulation, Iran

## Abstract

**Background:**

Midwives' stress can have negative consequences on their emotional state, burnout, and poor quality of midwifery care. This study aimed to determine the effectiveness of an adapted mindfulness-based stress management program on perceived stress and the emotional regulation of midwives.

**Methods:**

The study was a parallel randomized clinical trial on the midwives working in general hospitals of Zanjan, Iran. In this study, 121 midwives registered to participate based on the census sampling method were screened using a cut point of ≥ 28 in the Perceived Stress Scale (PSS). From the initial sample, 42 subjects had inclusion criteria assigned to two groups of control (n = 21) and intervention (n = 21) using online random allocation. The intervention group received an 8-week adapted mindfulness-based stress management program. This program emanates from the Kabat-Zinn's MBSR program, which has been adjusted according to the Iranian culture. The ANCOVA and repeated measure analysis of variance test were used to compare groups over time.

**Results:**

The results showed that the group intervention effectively affected perceived stress (*P* = 0.001) and difficulty in emotion regulation during the post-intervention period (*P* = 0.001). Moreover, the interventions were effective in emotion regulation (*P* = 0.003), but it was not effective on perceived stress (*P* = 0.125) at the 3-month follow-up.

**Conclusions:**

This adapted mindfulness-based program successfully reduced stress and increased emotion regulation strategies in midwives; however, the long-term outcomes of this treatment program need further consideration.

## Introduction

Stress is a prevalent phenomenon that has become more complex with the modernization of human life [1]. Perceived stress is defined as individuals’ reporting of situations as unpredictable, uncontrollable, and overwhelming [2]. The prevalence and causes of stress have been reported differently in health care workers [3–5]. Midwifery is a stressful profession, and environmental, background, and other factors exacerbate stress [6]. When midwives experience a traumatic event, they may experience feelings of guilt, shame, blame, isolation, helplessness, and despair, which make them a second victim [7]. A study showed that almost all midwives (97.1%) experienced traumatic birth events [8]. The prevalence of stress, anxiety, depression, and burnout among midwives is high [3]. In addition, midwives reported more stress beyond the nurses' colleagues due to less support, less autonomy and less clarity in role [9]. The results of a meta-analysis showed that about 71% of midwives in Iran have occupational stress [10].

Stress impairs decision-making and increases midwifery errors, resulting in increased mortality and morbidity in pregnant women and infants [11]. Also, stress reduces the quality of patient care [10], the correct and timely decision-making [12], and the ability of employees to be skilled and committed [13]. The conditions of midwives are stressful, although, they are less taught about stress management [14]. A qualitative study of midwives and nurses has shown that most of them had to vary emotional demands and deficits that need to be trained with emotion regulation (ER) skills [15]. Therefore, midwives need effective interventions to reduce stress, and increase the abilities of coping [8, 10, 16].

ER known as an ability to manage and regulate emotions (including stress) [17]. ER strategies also divided into maladaptive (e.g., suppression, experiential avoidance and rumination) and adaptive (e.g., acceptance, problem solving and reappraisal) [18, 19]. Individuals who used maladaptive ER strategies were at greater risk for emotional disorders [20, 21]. In return, adaptive ER strategies had a strong association with well-being [22]. Individuals have a different ability to ER [23]. Nevertheless, ER skills are learnable, and evidence has shown that training ER in health care can increase their ability to cope with stressors and negative emotions [24].

Despite the urgent need of this group of health care workers for effective interventions, there is a wide gap in the interventional studies in this area [25]. The two well-known interventions from this field include cognitive-behavioral stress management [e.g. 26, 27], and mindfulness based programs [e.g. 28–30]. Cognitive-behavioral stress management program commonly focus on psychoeducation, cognitive modification, and lifestyle change [31, 32]. Evidence supports the effectiveness of this program. For example, a 10-week cognitive-behavioral stress management leads to increase in perceived stress management competency, self-efficacy, and self-esteem 1 year after the intervention [26]. In addition, mindfulness-based stress reduction (MBSR) program caused stress reduction due to mindfulness, and self-compassion [33]. A meta-analysis showed MBSR interventions could moderately reduce stress, depression, anxiety, and distress [34].

Both cognitive-behavioral stress management and mindfulness-based programs are known to be effective in reducing midwives' stress. A cognitive-behavioral stress management programs effectively reduced midwives occupational stress rather than wait-lists [35]. Also, an adapted mindfulness based program leads to nurses and midwives' stress reduction and enhancing health, sense of coherence and resiliency [36]. However, our knowledge of the simultaneous use of a cognitive-behavioral program with mindfulness exercises is limited, while it seems to be able to both effectively improve emotion regulation and reduce stress. This study aimed to evaluate the effectiveness of adapted group mindfulness based stress management program to reduce midwives’ stress and improve their emotion regulation strategies.

## Methods

### Study design

The study was a parallel randomized clinical trial registered on the Iranian clinical trials website (IRCT20160608028352N6). The study was registered in Iranian Registry of Clinical Trials (https://en.irct.ir/trial/23028). The date of first registration was 08/01/2018. The sampling of the study lasted from January to May 2018.

### Participants and setting

**Fig. 1:**
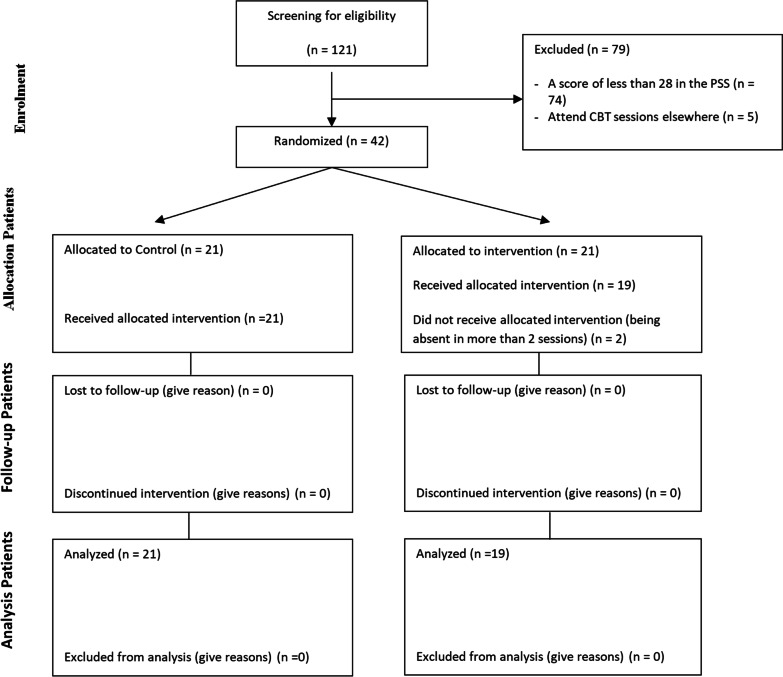
CONSORT flow diagram of participants

There are two governmental hospitals with obstetrics and gynecology centres in Zanjan that both of were selected for this study and the study population included all 121 midwives working in these centres. Sampling was done by census method, and all midwives working in hospitals were screened to participate in the study. Midwives were screened for stress with the Perceived Stress Scale (PSS). Also, all samples participated in a clinical interview conducted by a clinical psychologist to examine the inclusion and exclusion criteria. Eligibility criteria for participants included having a bachelor's degree or higher in midwifery, getting a score above 28 from PSS, having 1 year of experience in midwifery, not using cigarettes and drugs, not attending other counseling sessions in the last 2 months, lack of other stressful conditions in life such as divorce or death of a loved one, and not being pregnant. Participants who were reluctant to continue the study, or were absent for more than two sessions, or fill out the questionnaire incompletely, were excluded from the study. They were also excluded if they were diagnosed with mental disorders at any phase of the study. The study’s power with 0.05 errors, in the post-test and follow-up periods, was 1.00 and 1.00 for perceived stress and 1.00 and 0.87 for emotion regulation, respectively. From 121 midwives in the initial screening, 42 midwives had a score equal to or more than the cut-off point (score ≥ 28) of PSS and also reached the criteria for entering the study. After obtaining informed consent, the participants were assigned to group intervention program (n = 21) and control (n = 21) using the randomized design method, which was conducted through an online website https://www.randomizer.org. The control group participants were waiting to receive treatment sessions until the follow-up stage of the interventions. They did not receive any intervention during this time. Two participants in the intervention group were excluded because they were absent in more than two sessions (Fig. 1).

### Measures

In this study, two instruments were used to evaluate the outcome measures which include:

(1) The Perceived Stress Scale (PSS): the PSS is a 14-item self-report tool that has designed to evaluate perceived stresses over the past 10 weeks. The PSS has a Likert 5-point scale, which is graded from never (0) to always (4). Items 4, 5, 6, 7, 9, 10, and 13 are scored in reverse order. Cronbach's alpha coefficient in the original community of the scale provider was 0.85. The cut-point higher than 28 on this scale means high perceived stress. In an Iranian study, Cronbach's alpha was 0.76 [37, 38].

(2) The Difficulties in Emotion Regulation Scale (DERS): the DERS was designed in 2004 with 36 items to measure emotional disturbances and emotional self-regulation strategies [39]. Six subscales of this scale include non-acceptance of emotional responses, difficulty engaging in goal-directed behaviour, impulse control difficulties, lack of emotional awareness, limited access to emotion regulation strategies, and lack of emotional clarity. Higher scores on this scale indicate greater difficulty in regulating emotions. The response to this scale based on the Likert scale is from one to five. The scale has a total score for all questions and six scores related to the subscales. Cronbach's alpha of DERS in the original community of the scale provider was reported to be 0.93 and in Iran 0.90 [39, 40].

### Intervention

The mindfulness based stress management program that used in the present study was an adapted version of Kabat-Zinn's MBSR [41]. This protocol developed by Jalali and Aghaei 2016 to adapt with Iranian culture in titled "Mindfulness Based Cognitive-Behavioral Stress Management" [42]. This treatment program concentrates on psychoeducation, problem solving, and mindfulness exercises to address occupational stress. Preliminary evidence supports the effectiveness of this treatment program on dysfunctional attitudes and job affects in employees.

It was carried out in 8 sessions of 90 to 120 min once a week. Group discussions and exercises were organized in such a way that covered all cognitive, physiological, emotional, and behavioural factors of the work environment stress (Table 1). At the end of each session, assignments were given for the next week. The assignments addressed stress reduction due to the physiological, psychological, behavioural, and organizational consequences of midwives. Metaphors, allegories, and poetry were used to increase understanding of the content according to the adapted intervention program. Two expert in clinical psychology and midwifery counselling which well trained in mindfulness carried this group program. Supervisors guided the experts about manners of the sessions and reviewed assignments.

**Table 1:** The content and homework of adapted group mindfulness based stress management program

Session	Content	Homework
First	Pre-test and participants' acquaintance with each other and group rules The concept of perceived stress, physiological, psychological and behavioral outcomes of stress The concept of assessment and how to form triple assessments in a stressful situation	Identify stressors of life and work environment, demands and resources of work and life, study the signs and consequences of stress and workplace stress, how to evaluate different situations of life and work environment
Second	The concept of mindfulness and automatic guidance Practice eating raisins to explain the logic concerning mindfulness	Identify stressful situations and how to evaluate different work and life situations Pay attention to life situations and focus attention Practice eating raisins or any other similar exercise on a daily basis and record the resulting experiences
Third	The concept of staying in the present time, explaining the philosophical foundations of the past, present and future The concept of attention span Perform sitting meditation Practice focusing for a minute and staying in the present The concept of awareness of breathing Practice three-minute breathing awareness	Identify stressful situations and how to evaluate different work and life situations Practice one-minute and three-minute breathing awareness
Fourth	The concept of body meditation, the ability to combine the characteristics of mindfulness with gained experience The concept of the unity of mind and body Practice body assessment with the example of the elephant in the dark	Identify stressful situations and how to evaluate different work and life situations Practice three-minute breathing awareness and body assessment meditation
Fifth	The association of thoughts with emotions and behaviors Automatic thoughts, rumination Positive and negative emotions, the concept of organizational justice, sitting with thoughts and feelings and practicing it The concept of acceptance	Identify stressful situations and how to evaluate different work and life situations Complete an extended ABC form to identify the relationship between thoughts, emotions, behaviors, and body sensations Practice three-minute breathing awareness and body assessment meditation
Sixth	The concept of self- compassion, familiarity with compassionate meditation Practice loving meditation Non-judgmental mind and initiator mind The role of mindfulness in improving relationships, the impact of judgments on communication	Identify stressful situations and how to evaluate different work and life situations Practice three-minute breathing awareness, body assessment meditation, compassionate meditation, and loving meditation
Seventh	The concept of coping, coping styles with stress Efficient and inefficient styles Time management, allegory of rubble and fine and coarse sand, expression of awareness of activities in daily life and work environment	Identify stressful situations and how to evaluate different work and life situations Practice three-minute breathing awareness and body assessment meditation Emotion-oriented and problem-oriented strategies in stressful situations Rubble and coarse grains in life and work environment
Eighth	How to maintain changes in life and work environment, summarizing, reviewing last week's assignments and completing questionnaires	Practicing awareness of breathing Tuesday minute of and meditation inspection body

### Variables

The primary outcome of the study was the perceived stress, and the secondary outcome was difficulties in emotion regulation. The outcomes were evaluated by the PSS and DERS in three phases; before the intervention, in the last session, and 3 months after the last session.

### Statistical analysis

Chi-square and independent t-test were used for comparing demographic characteristics and dependent variables between the two groups. Kolmogorov–Smirnov test was used to verify the normal distribution of data and Levene’s tests to test the equality of variances. Repeated measure ANOVA was used to evaluate the changes of dependent variables from the pre-test to post-test and follow-up phases. One-way Analysis of Covariance (ANCOVA) was used to compare the dependent variables between the two groups adjusted for pre-test and demographic differences. The spouse's job and education were controlled as covariance or diffraction in ANCOVA due to the significant differences between the control and intervention groups (*p* < 0.05). The analysis was performed using SPSS-16, and a *p*-value less than 0.05 was considered to be statistically significant. The analyst who performed the statistical analysis was out of the study and was blind to all stages of the research.

### Ethical considerations

The study has complied with the ethical considerations related to clinical trials, and all methods were performed in accordance with the relevant guidelines and regulations of the Research Ethics Committee of Zanjan University of Medical Sciences. The Research Ethics Committee of Zanjan University of Medical Sciences approved the protocol of the study with the code of ethics ZUMS.REC.1396.237. All participants were aware of the study guidelines and completed informed consent forms.

## Results

The mean (SD) of age in the intervention and control groups were 31.72 (6.84) and 32.64 (6.17) years, respectively. Also, the mean of work experience in the intervention and control groups were 6.89 (6.44) and 8.45 (6.05) years, respectively. There were no statistically significant differences between the intervention and control groups regarding the marital status, number of children, education, workplace departments, employment type, salary, homeownership, and having a vehicle. However, there were statistically significant differences between the two groups in terms of spouse's job (*p* = 0.001) and spouse's education (*p* = 0.026) (Table 2). Also, there were no statistically significant differences between the groups in outcome variables in the pre-test.

**Table 2:** Demographic characteristics of the participants in the two groups

Variable	Control group	MBSR	*P* value*
	N (%)	N (%)	
*Total sample*	21 (100)	19 (100)	
*Marital status*			
Single	2 (9.5)	6 (31.6)	0.120
Married	19 (90.5)	13 (68.4)	
*Children*			
No Children	10 (47.6)	14 (73.7)	0.248
One child	5 (23.8)	2 (10.5)	
Two children	6 (28.6)	3 (15.8)	
*Education*			
Undergraduate	20 (95.2)	17 (89.5)	0.596
MSc or higher	1 (4.8)	2 (10.5)	
*Departments*			
Childbirth block	10 (47.6)	12 (63.1)	0.802
Caesarean section	2 (9.5)	1 (5.3)	
Surgery room	1 (4.8)	0 (0.0)	
Emergency department	6 (28.6)	4 (21.0)	
Elective	1 (4.8)	1 (5.3)	
IVF	1 (4.8)	1 (5.3)	
*Employment type*			
Non fixed term contract	2 (9.5)	8 (42.1)	0.041
Fixed term contract	6 (28.6)	3 (15.8)	
Contractual	3 (14.3)	5 (26.3)	
Official (experimental)	1 (4.8)	1 (5.3)	
Official (definitive)	9 (42.8)	2 (10.5)	
*Spouse's occupation*			
Employee	19 (90.5)	7 (36.8)	0.001
Others	0 (0.0)	6 (31.6)	
Deceased	2 (9.5)	6 (31.6)	
*Spouse's education*			
Under the diploma	1 (4.8)	0 (0.0)	0.026
Associate degree	0 (0.0)	3 (15.8)	
Undergraduate	10 (47.6)	7 (36.8)	
Masters	7 (33.3)	1 (5.3)	
Ph.D.	1 (4.8)	2 (10.5)	
Deceased	2 (9.5)	6 (31.6)	
*Salary*			
Under 2 million Tomans	6 (28.6)	9 (47.4)	0.074
2 to 2.5 million Tomans	4 (19.0)	6 (31.6)	
2.5 to 3 million Tomans	7 (33.3)	0 (0.0)	
3 to 3.5 million Tomans	3 (14.3)	2 (10.5)	
4 million Tomans and more	1 (4.8)	2 (10.5)	
*Home ownership*			
Owner	16 (76.2)	12 (63.2)	0.293
Tenant	4 (19.0)	7 (36.8)	
Others	1 (4.8)	0 (0.0)	
*Having a vehicle*			
Owns	20 (95.2)	15 (78.9)	0.172
Doesn't own	1 (4.8)	4 (21.1)	

^*^Chi-square test

Perceived stress (Eta = 0.091, *p* = 0.149) and difficulty in emotion regulation scores (Eta = 0.077, *p* = 0.200) did not change significantly in the control group. At the same time, interventions reduced the total score of perceived stress (Eta = 0.374, *p* = 0.001) and perceived hopelessness (Eta = 0.264, *p* = 0.004) and improved perceived self-efficacy (Eta = 0.265, *p* = 0.004). The interventions had improved emotion regulation strategies (Eta = 0.264, *p* = 0.004). Midwives were more likely to accept their emotional responses after treatment (Eta = 0.210, *p* = 0.014), were able to perform goal-oriented behaviours in the face of a stressful situation (Eta = 0.228, *p* = 0.010), to access emotional strategies more easily (Eta = 0.396, *p* = 0.001), and to improve emotional clarity (Eta = 0.163, *p* = 0.041). However, interventions could not reduce impulse control (Eta = 0.069, *p* = 0.279) and did not increase emotional awareness (Eta = 0.031, *p* = 0.565) (Table 3).

**Table 3:** Dependent variables changes during three phases of study in the control and intervention groups

Group	Dependent variable	Mean (SD)			F	*P* value*	Eta
		Pre-test	Post-test	Follow Up			
Control	Perceived helplessness	13.71 (2.67)	12.19 (2.71)	13.00 (2.10)	2.74	0.077	0.120
	Perceived self-efficacy	16.29 (3.17)	15.95 (3.83)	14.90 (2.93)	1.32	0.278	0.062
	Total Perceived Stress Score	30.00 (3.34)	28.14 (4.11)	27.90 (3.51)	2.00	0.149	0.091
	Non acceptance of emotional responses	13.43 (5.45)	15.48 (4.59)	14.05 (3.12)	1.67	0.202	0.077
	Difficulty engaging in Goal-directed behavior	15.00 (4.83)	15.43 (3.64)	14.90 (4.19)	0.15	0.866	0.007
	Impulse control difficulties	16.00 (5.09)	16.81 (3.80)	15.81 (3.80)	0.48	0.623	0.023
	Lack of emotional awareness	14.43 (4.12)	15.62 (3.64)	16.33 (2.42)	2.85	0.070	0.125
	Limited access to emotion regulation strategies	20.95 (6.70)	23.71 (5.50)	22.43 (4.85)	1.89	0.165	0.086
	Lack of emotional clarity	9.19 (3.01)	10.14 (3.31)	10.76 (2.38)	2.00	0.149	0.091
	Total score of emotion regulation difficulties	89.00 (19.18)	97.19 (18.14)	94.29 (14.29)	1.68	0.200	0.077
Intervention	Perceived helplessness	12.68 (2.08)	9.63 (1.83)	10.68 (3.80)	6.44	0.004	0.264
	Perceived self-efficacy	16.84 (2.34)	13.84 (2.34)	14.53 (3.73)	6.48	0.004	0.265
	Total Perceived Stress Score	29.53 (2.65)	23.47 (2.54)	25.21 (6.69)	10.73	< 0.001	0.374
	Nonacceptance of emotional responses	13.95 (5.72)	10.58 (3.11)	11.26 (3.81)	4.77	0.014	0.210
	Difficulty engaging in Goal-directed behavior	14.11 (3.83)	12.00 (2.75)	11.74 (3.14)	5.30	0.010	0.228
	Impulse control difficulties	15.11 (4.12)	13.58 (2.89)	13.89 (3.71)	1.32	0.279	0.069
	Lack of emotional awareness	16.63 (4.83)	15.63 (2.67)	16.00 (2.52)	0.58	0.565	0.031
	Limited access to emotion regulation strategies	22.42 (6.98)	16.21 (3.69)	17.16 (5.37)	11.78	< 0.001	0.396
	Lack of emotional clarity	10.63 (3.82)	8.84 (1.57)	10.21 (2.97)	3.49	0.041	0.163
	Total score of emotion regulation difficulties	92.84 (23.28)	76.84 (11.66)	80.26 (17.96)	6.47	0.004	0.264

^*^Repeated measure Analysis of Variance

At the post-test phase, adjusted for the aforementioned variables, the interventions group reported higher scores on perceived stress and difficulties in emotion regulation than the control group (*p* < 0.05). At the follow-up phase, only the perceived helplessness component of PSS was significantly lower in the interventions group than the control group (*p* = 0.032). Moreover, the total score of emotion regulation difficulties were significantly lower in the interventions compared to the control group at the post-test (*p* < 0.001) and follow-up phases (*p* = 0.003). There were statistically significant differences between the two groups in terms of all components except for lack of emotional awareness at the post-test phase and lack of emotional awareness, lack of emotional clarity, and difficulty controlling impulses at the follow-up phase (Table 4).

**Table 4:** Dependent variables differences between the control and intervention groups adjusted for baseline variables

Phase	The dependent variable	Modified mean (Std. Error)		F	*P* value*	Eta
		Control	Intervention			
Post test	Perceived helplessness	12.07 (0.51)	9.77 (0.54)	9.33	0.004	0.206
	Perceived self-efficacy	16.23 (0.79)	13.53 (0.83)	5.39	0.026	0.130
	Total Perceived Stress Score	28.30 (0.96)	23.30 (1.01)	12.52	0.001	0.258
	Disapproving emotional responses	15.69 (0.75)	10.34 (0.80)	22.45	< 0.001	0.412
	Difficulty in carrying out purposeful behavior	15.36 (0.67)	12.08 (0.71)	10.67	0.003	0.250
	Difficulty in controlling impulses	16.60 (0.73)	13.81 (0.77)	6.49	0.016	0.169
	Lack of emotional awareness	15.93 (0.69)	15.28 (0.73)	0.38	0.538	0.012
	Limited access to emotional strategies	24.06 (0.88)	15.83 (0.93)	39.05	< 0.001	0.550
	Lack of emotional clarity	10.44 (0.58)	8.52 (0.61)	4.96	0.033	0.134
	Total score of emotion regulation difficulties	98.08 (3.18)	75.86 (3.36)	21.74	< 0.001	0.405
Follow-up	Perceived helplessness	12.96 (0.68)	10.73 (0.72)	4.97	0.032	0.121
	Perceived self-efficacy	14.97 (0.78)	14.45 (0.79)	0.22	0.642	0.006
	Total Perceived Stress Score	27.93 (1.19)	25.18 (1.25)	2.47	0.125	0.064
	Disapproving emotional responses	14.25 (0.80)	11.04 (0.85)	7.13	0.012	0.182
	Difficulty in carrying out purposeful behavior	14.81 (0.78)	11. 84 (0.82)	6.57	0.015	0.170
	Difficulty in controlling impulses	15.78 (0.81)	13.93 (0.86)	2.32	0.138	0.068
	Lack of emotional awareness	16.84 (0.47)	15.44 (0.49)	3.99	0.054	0.111
	Limited access to emotional strategies	22.98 (1.11)	16.56 (1.17)	14.94	0.001	0.318
	Lack of emotional clarity	11.05 (0.60)	9.89 (0.63)	1.68	0.204	0.050
	Total score of emotion regulation difficulties	95.70 (3.51)	78.70 (3.70)	10.46	0.003	0.246

^*^ANCOVA

## Discussion

In this study, the adapted group mindfulness based stress management program reduced the overall score of perceived stress and difficulty in emotion regulation. However, some of the subscales were not statistically changed at the post-test and follow-up phases.

The present intervention program was effectively reduced midwives’ stress, but despite its effects on reducing midwives’ perceived hopelessness, it failed to maintain their self-efficacy over a 3-month period. Present program targets stress's cognitive, emotional, behavioral, and physiological aspects through mindfulness, acceptance, self-forgiveness, compassion, being present in the moment, breathing awareness, meditation, and problem-solving strategies.

In terms of reducing perceived stress, the results of the present study are in line with other studies. For instance, stress inoculation training had been effective on midwives’ occupational stress in 1-month follow-up [27]. Also, there are considerable evidences regarding efficacy of mindfulness based cognitive therapy on stress, anxiety and depression [43–45]. Moreover, an 8-session mindfulness treatment was effective on midwives' stress immediately after intervention and at 4 to 6-month follow-up in another study [28]. Both of the above studies showed that psychological interventions focused on stress have been able to reduce the occupational stress of midwives, and the stability of these effects remains in the follow-up periods. The results of the present study are also consistent with these studies; in this way, both in the post-test and in the follow-up, it has been able to reduce the perceived stress of midwives. However, a study in Iran showed that stress management was effective immediately after the intervention but not in 1 month later [35]. The reason for the inconsistency of our study with the last-mentioned study may be that their study was held in the form of a 2-day psycho-educational workshop, and the entire duration of the training course lasted 4 h. Our follow-up period has been 3 months, during which the treatment interventions was able to maintain the therapeutic effects on reducing perceived stress. The purpose of this study, unlike previous studies, was to focus on perceived stress rather than occupational stress because occupational and job stress is a construct that is partially influenced by workplace and interpersonal concomitances and could change indirectly through a change in the conditions. Nevertheless, perceived stress has the most to do with peoples’ health and psychological state and is less affected by the environment. Changes in perceived stress can have far better effects on midwives’ mental and physical health. This is the first study on midwives in a clinical trial with a control group. The long-term effects of this intervention on health care workers have been less studied. In the present study, interventions was effectively reduced perceived stress and its’ subscales during the 3-month follow-up but compared to the control group, after 3 months, its’ effects on perceived self-efficacy decreased but on perceived hopelessness maintained. Although the 3-month follow-up is not a long-term period, there is hope that the effect of this intervention program on perceived self-efficacy will become more apparent over time.

The present intervention program was effective on the emotion regulation of midwives immediately after the intervention and in the 3 months follow-up, but had no significant effect on dimensions of controlling impulses, emotional awareness, and clarity. The effectiveness of this intervention in regulating the emotion of midwives is in line with other studies that have been done in this field [46, 47]. FMRI-based evidence shows that 8-week MBCT intervention significantly improved executive control and emotion regulation [48]. Heredia et al. also reported that although the MBSR has led to psychological well-being and improved emotional regulation, it has not improved performance of attention [46]. Given that impulse control difficulties, lack of emotional awareness, and clarity are fundamentally related to the attention, and results may be considered inconsistent with the underlying theory. There is much confirmatory evidence regarding the role of attention in improving emotional regulation. There are various emotion control strategies, and it is still unclear that MBSR has the most impact through which one of these strategies [49]. The functional Magnetic Resonance Imaging (FMRI) evaluation showed that among five emotional regulation strategies: situation selection, situation modification, attention deployment, non-judgmental awareness, reappraisal, and facilitating opposed action tendencies; mostly MBSR influenced the emotional regulation by the attention deployment [50].

The lack of effectiveness of the intervention on variables of impulse control difficulties, lack of emotional awareness and clarity in the present study may be due to different reasons. First the impulse control ability is a skill that is in the last cycle of emotion regulation feedback, and the effectiveness of this mindfulness based program on it require its’ impact on all previous cycles. Second, the emotional awareness subscale is one of the subscales that in new studies of factor analysis of DERS cannot explain the total variance load and its’ items are not properly designed to measure this construct. Third, the results related to emotional clarity are unexpected that need to be further studied. Ultimately, it should be noted that this adapted program is not specific psychotherapy for emotion regulation, but this study sought to evaluate its’ effectiveness on possible improvement in emotion regulation strategies. The strengths of this study are that in our knowledge this is the first clinical trial conducted on the effect of this adapted mindfulness based program on midwives. Also, it emphasizes perceived stress instead of occupational stress. For the first time, it has shown the improvement of emotion regulation strategies under the MBSR. The study results could be generalized to Iranian population of working midwives in governmental hospitals. The limitations of this study included the absence of a placebo group and long-term follow-up. In addition, the adherence to treatment program did not assessed systematically. Furthermore, due to the lack of blinding and credibility assessment all results need to be interpreted with notice.

## Conclusion

Based on the results of this study, it seems that the adapted group mindfulness based stress management program reduces the perceived stress and improves the emotional regulation in midwives in a short period. This program seems to be effective in reducing stress and increasing individual and workplace emotion regulation strategies; therefore, this intervention can be used for both prevention and treatment in midwives. However, more studies are suggested for assessing its longer effects. Effects of present intervention program on some dimensions such as impulse control difficulties, lack of emotional awareness and clarity, which are related to the attention, require more complex and time-consuming interventions.

## Data Availability

The datasets analysed during the current study are available from the corresponding author on reasonable request.
